# Case report: A *de novo* ERBB3 mutation develops in a gallbladder cancer patient carrying BRCA1 mutation after effective treatment with olaparib

**DOI:** 10.3389/fonc.2023.1078388

**Published:** 2023-03-09

**Authors:** Jing-Xiao Yang, Zi-Yao Jia, Fa-Tao Liu, Wen-Guang Wu, Xue-Chuan Li, Lu Zou, Huai-Feng Li, Fei Zhang, Run-Fa Bao, Shu-You Peng, Wan Yee Lau, Yun Liu, Mao-Lan Li, Ying-Bin Liu

**Affiliations:** ^1^ Department of Biliary-Pancreatic Surgery, Renji Hospital Affiliated to Shanghai Jiao Tong University School of Medicine, Shanghai, China; ^2^ Shanghai Cancer Institute, Shanghai, China; ^3^ State Key Laboratory of Oncogenes and Related Genes, Shanghai, China; ^4^ Shanghai Key Laboratory of Biliary Tract Disease Research, Shanghai, China; ^5^ Shanghai Research Center of Biliary Tract Disease, Shanghai, China; ^6^ Department of General Surgery, Xinhua Hospital Affiliated to Shanghai Jiao Tong University School of Medicine, Shanghai, China; ^7^ Department of General Surgery, The Second Affiliated Hospital of Zhejiang University School of Medicine, Hangzhou, Zhejiang, China; ^8^ Faculty of Medicine, The Chinese University of Hong Kong, Prince of Wales Hospital, Shatin, Hong Kong, Hong Kong SAR, China

**Keywords:** gallbladder cancer, dual targeted drugs therapy, olaparib, drug resistance, conversion therapy

## Abstract

**Background:**

Gallbladder cancer (GBC) is highly lethal and resistant to most chemotherapeutic drugs. GBC was reported to carry multiple genetic mutations such as TP53, K-RAS, and ERBB2/3. Here, we unexpectedly identified a patient with GBC harboring germline BRCA1 p.Arg1325Lys heterozygous mutation. We sought to determine if olaparib, the poly ADP-ribose polymerase inhibitor (PARPi) commonly treated for BRCA mutation, can inhibit cancer development *via* a therapeutic trial on this patient.

**Case presentation:**

The patient received GBC R0 resection after an 8-week olaparib treatment. After surgery and 6-month follow-up treatment with olaparib, the patient’s blood carbohydrate antigen 19-9 (CA19-9) level declined from 328 to 23.6 U/ml. No recurrence in CT scanning was observed, indicating a disease-free survival of 6 months with conventional therapy. Two months later, CT examination and CA19-9 level showed cancer relapse. A blood biopsy revealed a new ERBB3 p.Gly337Arg mutation. GBC cell lines ectopically expressing BRCA1 p.Arg1325Lys together with ERBB3 p.Gly337Arg mutations were challenged with olaparib and/or afatinib, an ERBB2/3 inhibitor. The dual mutation cells were more responsive to the combined olaparib with afatinib than a single drug in the cell proliferation assay.

**Conclusion:**

Olaparib is effective in a GBC patient with a BRAC1 mutation. The efficacy of olaparib and afatinib in both cultured BRAC1 and ERBB3 mutation cell lines suggests that a combined regimen targeting BRCA1/2 and ERBB2/3 mutations may be an optimal strategy to treat GBC patients who carry both gene mutations.

## Introduction

Gallbladder cancer (GBC) is the most common type of biliary tract malignant tumor and resists most chemotherapeutic drugs (1). The incidence of gallbladder cancer in different regions varies, as it depends on multiple cancer risk factors, including geographical region, gender, age, and food intake (2). According to the database of the International Agency for Research on Cancer (IARC) Globocan in 2020, there were an estimated 115,949 new cases of GBC worldwide (contributing 0.6% of new cases, ranking 25th out of 36 cancers in 185 countries), and gallbladder cancer contributes to 0.9% of all cancer deaths annually.

Although divergent therapeutic approaches are currently available to treat GBC patients, surgery is still the most acceptable and effective means. However, a fairly large proportion of patients missed the opportunity of surgical resection due to the lack of early typical symptoms, thus accepting alternative non-surgical treatment including chemo- and/or radiotherapy. In order to improve early diagnosis, considerable efforts have been made to develop detecting technologies with minimally invasive approaches. For example, liquid biopsies containing circulating tumor cells (CTCs) and circulating tumor DNA (ctDNA) from the blood have received significant attention in the assistance of clinical diagnosis. The high sensitivity of these techniques allows us to reveal tumor genetic features with aberrant gene expressions and gene mutations, thus providing great value for early diagnosis and therapy.

A plethora of research evidence has established the paradigm that altered expressions of tumor oncogenes and/or suppressor genes drive tumor cell proliferation and transformation in tumor development. Elevated or mutated genes including ERBBs, TP53, EGF, CYP7A1, and CCR5 emerge to regulate a variety of human cancer development (1). For instance, mutation of BRCA1, a tumor suppressor gene, has been shown to be intimately associated with the formation of familial breast and ovarian cancer (3). Olaparib, a Food and Drug Administration (FDA)-approved drug targeting BRCA1 and BRCA2 (BRCA1/2) mutations, functions to inhibit poly ADP-ribose polymerase inhibitor (PARPi). In clinical trials, PARPi has improved outcomes of BRCA1/2 mutated cancer patients including breast, ovarian, pancreatic, and prostate cancers (4). While ERBB2/3 mutation was previously discovered to mediate the malignancy of GBC, we still lack sufficient knowledge on other gene mutations and the role of the co-existence of ERBB2/3 mutation with BRAC1/2 mutation in the malignant transformation of patients with GBC. Here, we reported that a gallbladder cancer patient with BRCA1 p.Arg1325Lys mutation responded well to olaparib, but 6 months later, the patient exhibited cancer relapse and developed ERBB3 mutation. Tumor cell lines carrying BRCA1 and ERRB3 mutations were responsive to olaparib and afatinib, suggesting that a combined regimen targeting BRCA1/2 and ERBB2/3 mutations may be an optimal strategy to treat GBC patients who carried both gene mutations.

This study is a case report and agreed with the principles of the CARE guidelines (5).

## Methods

### Clinical information

**Figure 1:**
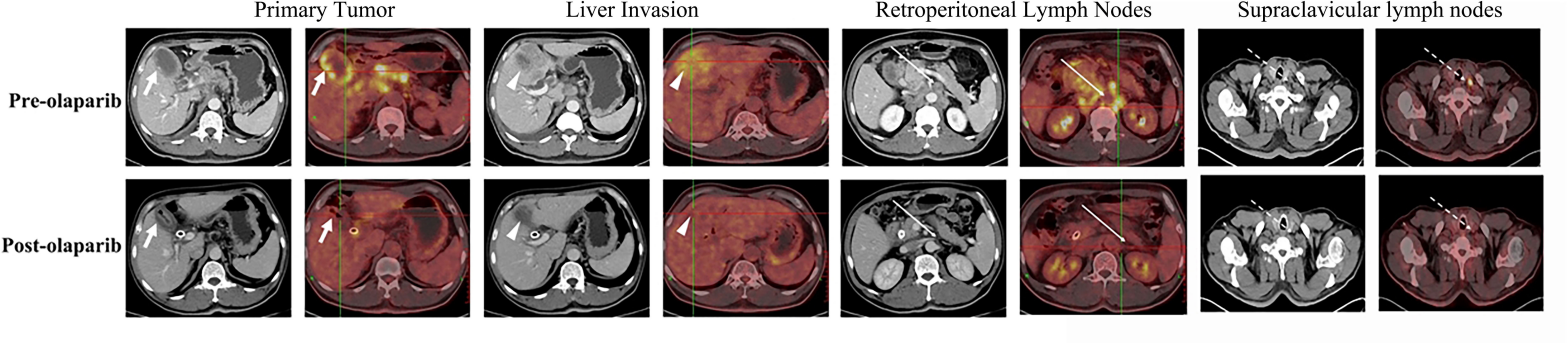
Representative pre- (upper row) and post-olaparib (lower row) treatment showing radiologic evidence in a 52-year-old man with gallbladder cancer harboring BRCA1 mutation. After 2 months of olaparib treatment, extensive metastatic lesions that involved the primary tumor (white arrow) and liver invasion (white arrowheads) became markedly reduced, while the retroperitoneal lymph nodes (long white arrows) and left supraclavicular lymph nodes (long white dotted arrows) disappeared when compared with those in the pre-olaparib treatment CT and PET/CT images.

The patient provided informed consent before clinical examinations. A 52-year-old man diagnosed with gallbladder cancer was recruited who initially expressed right upper quadrant pain and obstructive jaundice. In the intravenous contrast-enhanced CT and positron emission tomography (PET)/CT, the images showed gallbladder cancer with liver invasion (**Figure 1**). In addition, the images contained enlarged retroperitoneal and left supraclavicular lymph nodes. Laboratory tests showed abnormal liver function, with an alanine transaminase level of 241 U/L and an aspartate aminotransferase level of 119 U/L. The levels of total bilirubin and direct bilirubin were elevated to 80.4 and 64.6 μmol/L, respectively. Serum tumor biomarkers revealed an elevated level of carbohydrate antigen 19-9 (CA19-9) to 328  U/ml (reference range, <39 U/ml). The patient was evaluated to have unresectable locally advanced gallbladder cancer finally.

### Treatment

**Figure 2:**
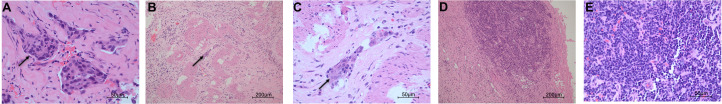
Histopathological examination of the specimens in pre- and post-olaparib treatment. **(A)** The cells (black arrow) from the infiltrating liver in pre-olaparib treatment are markedly pleomorphic with enlarged irregular nuclei (hematoxylin and eosin; magnification ×400). **(B, C)** After treatment with olaparib, only a very limited amount of cancer cells (black arrow) remained, and intense fibrosis developed (magnification ×100 in panel B and ×400 in panel **(C)**. **(D, E)** The resected lymph node was negative for malignancy (magnification ×100 in panel **D** and ×400 in panel **E**).

An endoscopic metal biliary endoprosthesis (EMBE) was engaged first to release the biliary obstruction and the needle biopsy from the liver confirmed adenocarcinoma with gallbladder cancer origin (**Figure 2A**). After EMBE, liver function improved significantly, and alanine and oxalacetic transaminases decreased to 61 and 40, respectively. Total bilirubin and direct bilirubin decreased to 27.4 and 10.4, respectively. Jaundice basically subsided.

The biopsy samples from liver metastasis were analyzed using the next-generation sequencing (NGS) method. A germline BRCA1 p.Arg1325Lys heterozygous mutation was detected and further confirmed by Sanger sequencing analysis. Subsequently, olaparib at 300 mg twice daily was given orally. The patient demonstrated remarkable clinical improvement with moderate adverse reactions such as nausea, diarrhea, or anemia in a course of 8-week treatment. CT and PET/CT exhibited favorable responses of the tumor to the treatment (**Figure 1**). The CA19-9 levels declined to 23.6 U/ml. His liver function levels, including alanine aminotransferase, aspartate aminotransferase, direct bilirubin, and indirect bilirubin, continued to improve. The tumor can be resected surgically after evaluation. As a result, the patient received radical surgery for the removal of the gallbladder, liver segments 4B and 5, common bile duct, and extended lymph node clearance. Histological examination of the resected surgical specimen showed cancer-free margins. All the resected lymph nodes were negative for metastasis. Thus, an R0 resection was achieved following olaparib treatment (**Figure 2**). Liquid biopsy with blood ctDNA after surgery showed the genetic signature of BRCA1 p.Arg1325Lys mutation. Following surgery, the patient kept receiving olaparib treatment at 300 mg twice daily for 6 months with mild adverse reactions similar to those described earlier at a local hospital. According to our follow-up, no tumor progression was found on the patient’s examinations during the medication period.

### Genetic mutation studies

Two months later with no drug treatment, the patient visited our hospital again and displayed a poor quality of life. The CA19-9 levels of this patient showed an elevation to 38.4 from 26.3 U/ml, and tumor regrowth was detected by CT scan images (not shown) in a local hospital, indicating cancer relapse. According to ctDNA analysis from blood biopsy, a new ERBB3 p.Gly337Arg mutation was found. There was compelling evidence revealing the development of some gene mutations such as RAD51C (6) and Abcb1a after treatment with PARPi (7). In addition, our previous studies demonstrated that ERBB3 mutation mediates gallbladder cancer proliferation and progression (8, 9). Given this result, we evaluated the patient’s general condition and devised the therapy strategy to add ERBB3 mutation inhibitors for combination treatment (olaparib plus afatinib). During the supportive treatment in order to attenuate intestinal obstruction, the patient exhibited cachexia and multiple organ functional failure with a performance status (PS) score of 4 out of 5. The patient and his family eventually chose to return to the local hospital for supportive treatment. According to our follow-up, the patient’s general condition continued to deteriorate and never improved. The patient died in 43 days, missing the opportunity of the combined therapy.

### *In vitro* experiments

**Figure 3:**
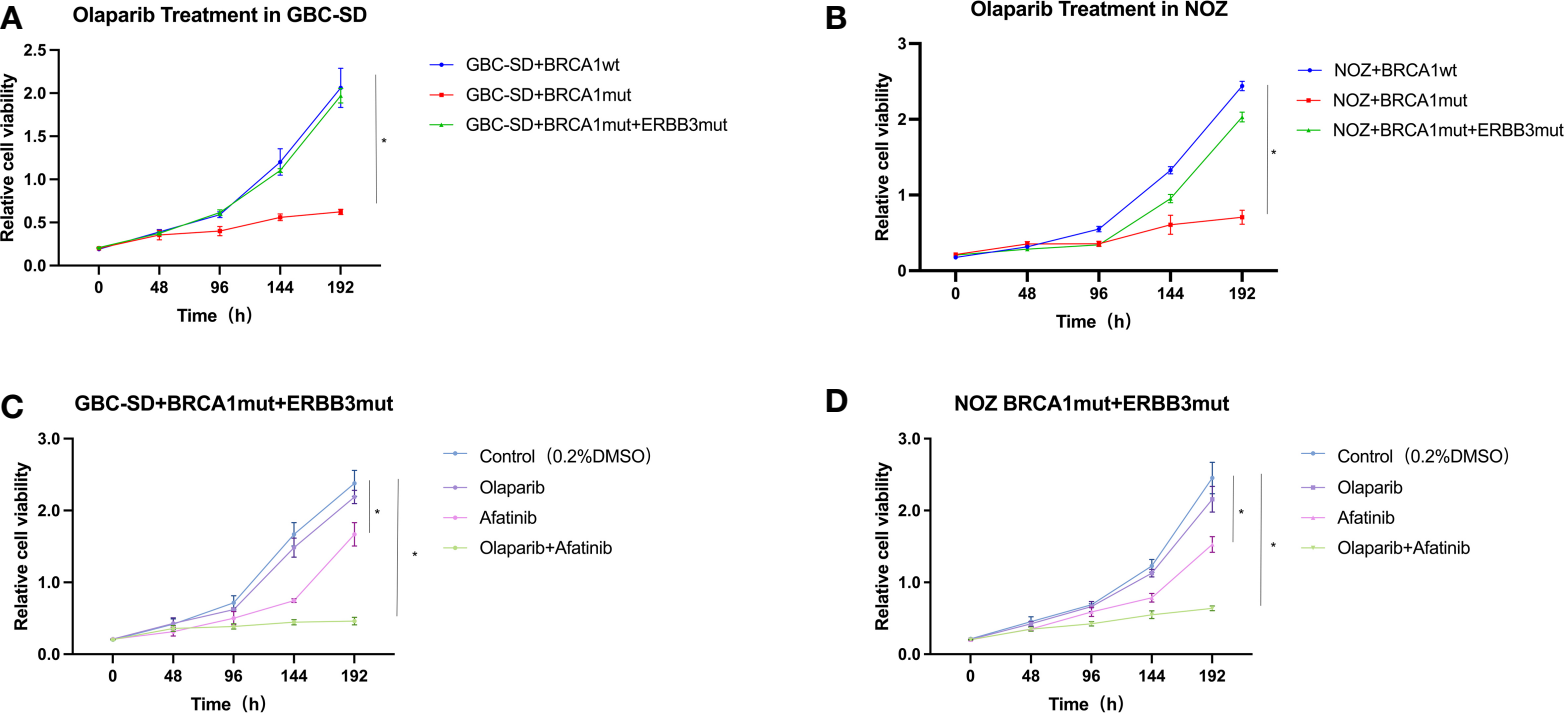
Cell viability of GBC-SD and NOZ cells expressing BRCA1 p.Arg1325Lys and ERBB3 p.Gly337Arg mutation. **(A)** GBC-SD cells expressing BRCA1 wild type (wt) or BRCA1 p.Arg1325Lys (mut) or both BRCA1 p.Arg1325Lys (mut) and ERBB3 p.Gly337Arg (mut) responded to olaparib (20 μM) over 192 h. **(B)** NOZ cells expressing both BRCA1 and ERBB3 wt or BRCA1 p.Arg1325Lys and ERBB3 p.Gly337Arg mutation responded to olaparib (20 μM) and afatinib (10 μM). **(C)** GBC-SD cells expressing both BRCA1 p.Arg1325Lys and ERBB3 p.Gly337Arg mutation responded to olaparib and afatinib. **(D)** NOZ cells expressing both BRCA1 p.Arg1325Lys and ERBB3 p.Gly337Arg mutation responded to olaparib and afatinib.

To examine if our early new therapeutic plan on the patient is reasonable and effective, we sought to use cultured cells exposed to olaparib and afatinib. Both wild-type and mutation constructs with single BRCA1 mutation and double BRCA1 and ERBB3 mutations were established in GBC cell lines GBC-SD and NOZ. After these cells stably expressed these genes, cell viability was analyzed in the presence of olaparib and/or afatinib. The results showed that GBC-SD cell lines with only BRCA1 p.Arg1325Lys mutation were more sensitive to olaparib when compared with the cells expressing the wild type (**Figures 3A, B**). However, cells with dual mutations of BRCA1 p.Arg1325Lys and ERBB3 p.Gly337Arg remarkably exhibited olaparib resistance (**Figures 3A, B**). Nevertheless, when afatinib was added, the sensitivity of these mutation cells to both these drugs resumed, and the effect of combined therapy was stronger than that of a single drug (**Figures 3C, D**). Similar results were obtained in colony formation and EdU assays of olaparib and/or afatinib on GBC cell lines ectopically expressing BRCA1 p.Arg1325Lys together with ERBB3 p.Gly337Arg mutations (**Supplementary Material Figures 1, 2**). The results suggest that the dual-targeted therapy may be more effective in gallbladder cancer patients with both BRCA1 and ERBB3 mutations than a single drug treatment.

### Statistical method

For comparisons of two groups, Student’s t-test was applied if no significantly different variances were present; otherwise, the Mann–Whitney test was used p < 0.05 was considered statistically significant.

## Discussion

While BRCA1/2 mutation has frequently been identified in a wide spectrum of cancers, including breast (10), ovarian (11), pancreatic (12), and prostate (13) cancers, BRCA1 p.Arg1325Lys has not been reported in GBC. This is the first time to demonstrate that BRCA1 p.Arg1325Lys exists in GBC.

In concert with the sensitivities of mutated BRCA1/2 cancer cells to PARPi, the patient responded well to olaparib, the first line of targeted therapy approved by the FDA for BRCA1/2 mutated cancers (14). After treatment with olaparib, CT and PET/CT showed dramatic restriction of the tumor. Histopathological examination also showed only a few remaining tumor cells with no lymph node metastasis. The blood levels of CA19-9 declined from 328 U/ml to the normal range. All the results demonstrated favorable responses to BRCA1 mutation-targeted therapy. Although it is one case reported with BRCA1/2 mutation in GBC, the favorable clinical outcome has encouraged us to extend our research in clinical trials with GBCs expressing BRCA1/2 mutation in the near future.

Accumulating evidence indicated that cancer cells harboring BRCA1/2 mutation are defective in double-strand DNA break (DSB) repair, and these cells are sensitive to PARP inhibitors (15–18). However, prolonged treatment with PARPi can eventually lead to the occurrence of drug resistance. At present, at least four distinct molecular mechanisms have been documented to explain PARPi resistance (19): i) increased ability to export drugs, which could be caused by overexpression of drug-efflux transporter genes (Abcb1a and Abcb1b encoding for MDR1/P-gp and Abcg2) in cancer cells (7), ii) increased activity and level of PAR chains through loss of PARG (20) or PARP1 mutations that diminish trapping of protein on DNA (21), iii) increased homologous recombination (HR) (22), and iv) replication fork protection. For instance, there are defects of SMARCAL1, ZRANB3, and HLTF that mediate double DNA chain fork, MRE11-dependent degradation of nascent DNA in BRCA1/2-deficient human cell lines (23). In addition, other mechanisms that downregulate DNA repair pathways such as overexpression of histone methyltransferases EHMT1/2 have also been reported to mediate PARPi resistance (24, 25). It is worthwhile to further decipher the molecular mechanisms underpinning the acquired resistance to olaparib in our case.

The safety of PARPi has received significant attention, though no obvious adverse and unanticipated events were observed in this patient. Adverse events (AEs) may result in the termination of PARPi treatment when AE has threatened the patients’ life. PARPi has a high risk of serious AE (SAE)- and AE-related discontinuation of treatment compared with placebo (26). This suggests that in the clinical application of PARPi, drug safety and AE should be particularly taken into account.

The combined therapy of PARPi with a DNA damage repair blocker such as topoisomerase I poison inhibitor was reported to sensitize tumor cell death and improve patient prognosis (27). In addition, a conjunction treatment of PARPi with anti-angiogenesis and anti-immune checkpoint drugs was also practiced in the clinical trials. However, the underlying mechanisms of these treatments remain to be clarified. Here, we found that the patient developed a new gene mutation with ERBB3 p.Gly337Arg when the disease did not respond to olaparib. It remains to be investigated if ERBB3 mutation is mechanistically associated with BRCA1 mutation. Nevertheless, our results with GBC cultured cell lines, which ectopically expressed both BRCA1 p.Arg1325Lys and ERBB3 p.Gly337Arg, developed resistance to olaparib. In contrast, single BRCA1 p.Arg1325Lys-mutated cells failed to grow in the presence of olaparib. Thus, dual inhibitors with olaparib and afatinib recapitulated the sensitivity of these cells to decreased cell growth, offering effective means to treat both mutations in GBC.

Compared with other previous cases of olaparib in the treatment of BRCA1 mutant gallbladder cancer (28, 29), the progression-free survival of the patient in our case was similar, but the mutation sites were different, which further demonstrated the feasibility of olaparib in the treatment of BRCA1 mutant gallbladder cancer. Different from other cases, the patient in this case was first treated with olaparib to shrink the tumor and successfully achieve R0 resection, thus achieving conversion therapy, which also provides a new idea for the surgical treatment of gallbladder cancer. In addition, this patient developed a new gene mutation with ERBB3 p.Gly337Arg, which was also one of the possible reasons for the progression of the disease. We also verified the effectiveness of dual-targeted drug therapy to a certain extent through an *in vitro* experiment, providing some ideas and references for similar situations that may occur in the future.

In summary, we reported that a patient with GBC harboring BRCA1 mutation initially responded well to olaparib. After a 6-month course of treatment, the patient developed an additional ERBB3 mutation, which led to the failure of the PARPi therapy. A dual BRCA1/2 and ERBB2/3 mutation-targeted therapy may suggest a benefit to GBC patients who carry both mutated genes.

## Data availability statement

The raw data supporting the conclusions of this article will be made available by the authors, without undue reservation.

## Ethics statement

Ethical review and approval was not required for the study on human participants in accordance with the local legislation and institutional requirements. The patients/participants provided their written informed consent to participate in this study. Written informed consent was obtained from the [individual(s) AND/OR minor(s)’ legal guardian/next of kin] for the publication of any potentially identifiable images or data included in this article.

## Author contributions

(I) Conception and design: YL, M-LL and Y-BL. (II) Administrative support: M-LL, Y-BL, S-YP and WL. (III) Provision of study materials or patients: Y-DZ, W-GW, X-AW, H-FL, FZ and R-FB. (IV) Collection and assembly of data: Z-YJ, J-XY, X-CL and LZ. (V) Data analysis and interpretation: F-TL, Z-YJ, J-XY, X-CL and LZ. (VI) Manuscript writing: all authors. All authors contributed to the article and approved the submitted version.
