# Supplementary figures and images for: Case report: A de novo ERBB3 mutation develops in a gallbladder cancer patient carrying BRCA1 mutation after effective treatment with olaparib
Source: Front Oncol. 2023 Mar 9;13:1078388. doi: 10.3389/fonc.2023.1078388 (PMC10034383; doi:10.3389/fonc.2023.1078388)

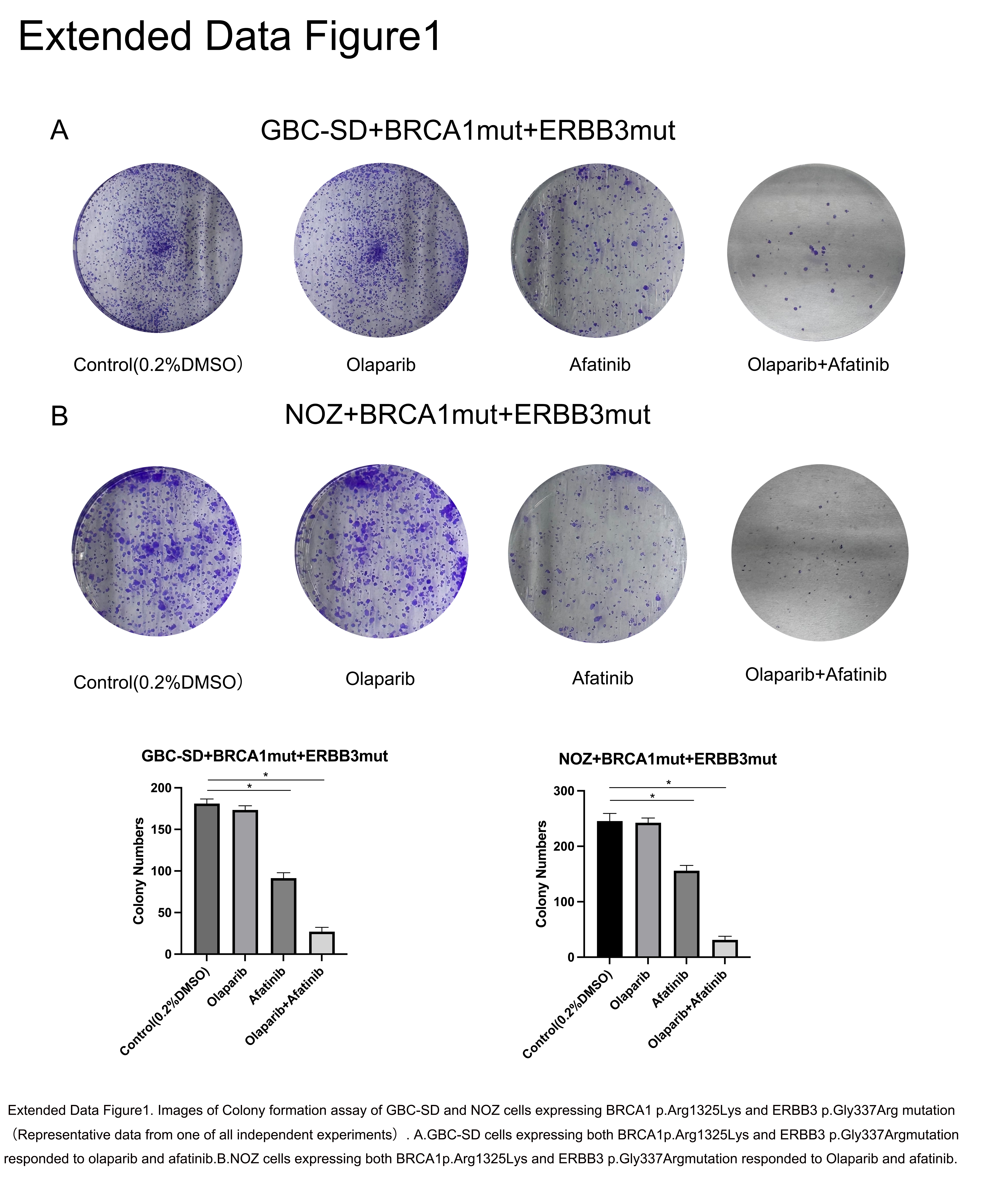

Supplement: Supplementary file 1 [file Image_1.jpeg]

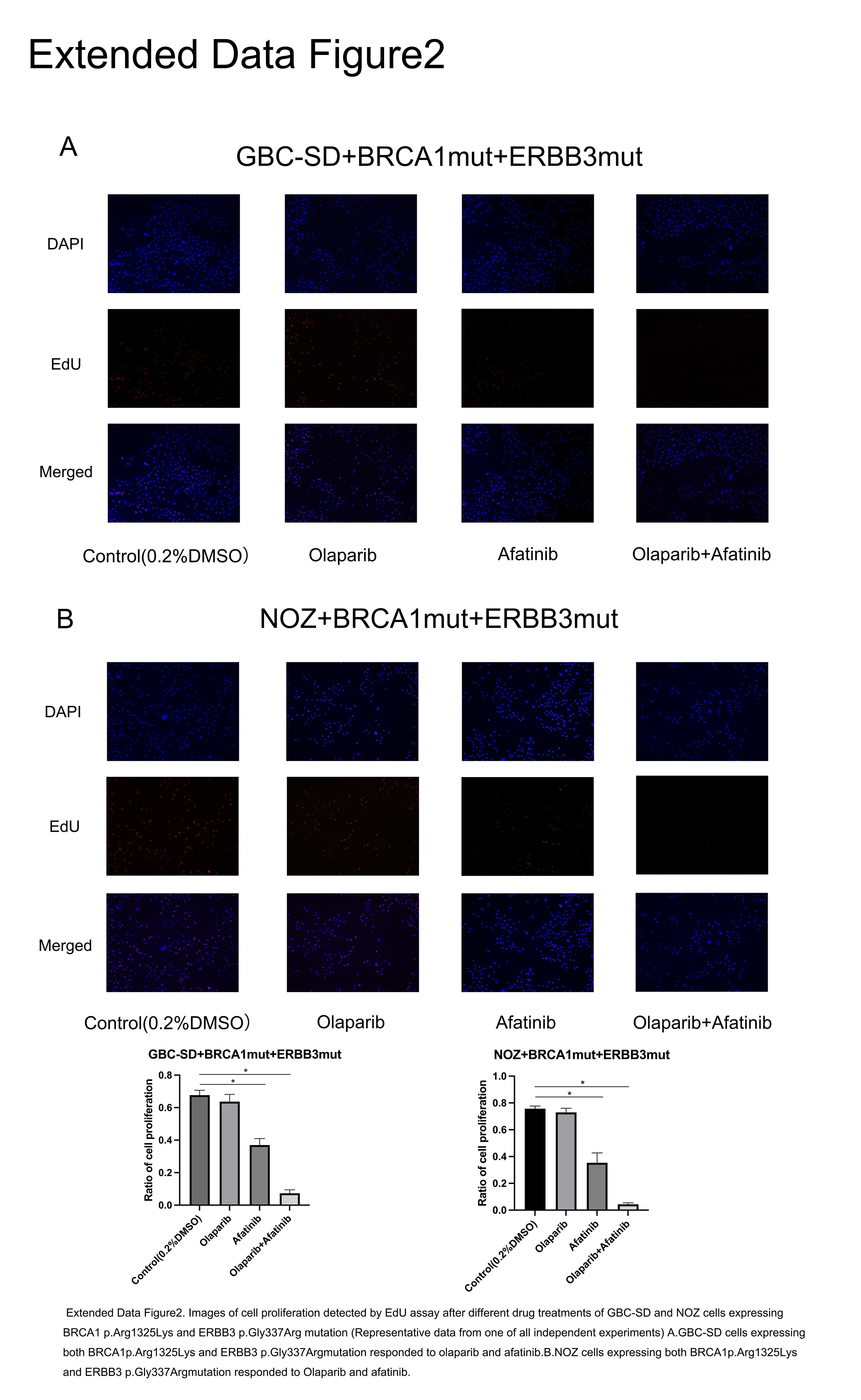

Supplement: Supplementary file 2 [file Image_2.jpeg]
